# Adoption and impact of improved *teff* varieties adoption on food security: Micro level evidence from North Eastern Amhara Regional state, Ethiopia

**DOI:** 10.1371/journal.pone.0291434

**Published:** 2023-09-20

**Authors:** Kindye Ayen Tilaye, Bosena Tegegne Delele, Mohammed Aman Ogeto

**Affiliations:** 1 Sekota Dry Land Agricultural Research Center (SDARC), Sekota, Ethiopia; 2 BahirDar University, College of Agriculture and Environmental Science, BahirDar, Ethiopia; 3 Haramaya University, College of Agriculture and Environmental Science, Haramaya, Ethiopia; CRIDA: Central Research Institute for Dryland Agriculture, INDIA

## Abstract

This study investigates the adoption and impact of improved teff varieties (ITVs) on food security in North Eastern Amhara Region, Ethiopia. The result of binary probit model shows that the adoption decision of improved teff varieties is determined by total family size measured in adult equivalent, off-farm income, number of traders known by farmers, variety information from development agents and research center affects positively and distance of farmer’s residence from kebele office of agriculture negatively Endogenous switching regression (ESR)model was employed to evaluate the impacts of adoption of improved teff varieties on food security. The output from ESR asserted that had the adopter households decided not to adopt, their average daily calorie intake and annual food consumption expenditure per adult equivalent would have decreased by 417.908 kilocalorie and 1096.509 Ethiopian Birr(37.8.41 dollars) respectively. Thus, policies and development endeavors targeting to realize food security should facilitate the adoption of ITVs.

## 1. Introduction

Poverty reduction and food security improvement in sub-Saharan Africa have been the major national and international development issues. The agricultural sector contributes a line share to food and nutrition security, as well as economic growth in many developing countries, and takes up 65% of the employment force and 32% of gross domestic product in Sub- Sahara Africa [1].To reverse this recurrent problem of food insecurity agricultural development and pro poor policies through extension and research investment endeavors have been implemented to maximize outputs and productivity of major staple crops grown by resource-poor smallholder farmers [2].

Well-developed and introduced agricultural technologies play an important role in increasing agricultural production and productivity, smallholder farm household income thereby to achieve food security, and in reducing poverty [2–7].

*Teff* is one of strategic food security cereal crop which plays a paramount role in achieving food security and enhancing income of households in most parts of Ethiopia. *Teff* [*Eragrostis teff*) is a small cereal grain which is indigenous to Ethiopia. In Ethiopia out of the total grain crop area, 80.71% was under cereals. *Teff* accounts 23.85% of the grain crop area and in terms of quantity of production; cereals contributed 87.48% of the grain production and *teff* account 17.26% of the total grain production. T*eff* is the most dominant cereal crops in Ethiopian agriculture. About 5.28 million tons of *teff* was produced from 3.02 million hectares of land in the main Meher season [8]. *Teff* has special merits such as special value in the national diet, provides a major feed source, well adapted to different agro-ecological zone and, its ability to tolerate both under low moisture stress and high moisture conditions and performs better under the above two extreme environmental conditions [9, 10]. *Teff* is the most dominantly consumed crop among different crops grown in the country, with more than 50 million domestic consumers. Moreover, *teff* is a gluten-free, nutritionally rich with high levels of iron and calcium and has the highest amount of protein among cereals consumed in Ethiopia. Moreover, *teff* has low glycemic index which making it suitable for consumption by Type II diabetics, high in fiber content and worldwide due attention was given in recent years [11].Therefore, to maximize the production productivity of *teff*, the adoption of promising improved *teff* technologies play paramount role to attain food security and poverty reduction in the country. In this regard, national and regional research investment endeavors have been undertaken to develop and dissemination of several improved teff varieties towards rural smallholder farmers in Ethiopia. To increase the productivity of *teff*, 42 improved teff varieties (**ITVs**) were developed by federal and regional research centers by different breeding mechanisms and disseminated towards smallholder farmers in the study area in the past 6 consecutive years [5].

A large volume of empirical literatures had been documented on the impact of improved agricultural technology adoption on food security and poverty reduction in Ethiopia. However, extant research conducted in evaluating the impact of improved technology on food security and poverty reduction in Ethiopia focuses primarily on other crops other than *teff* [2–7].

A few number of studies have been conducted on the impact of ITVs in different parts of the country and elsewhere, for example, [12–15]. However, all of these studies measure the impact of improved *teff* varieties limited to crop yield and crop income, which is the accessible dimension of food security. However, adequate supply of food at the national or international level by itself cannot be a guarantee for household level food security status of smallholder farm households [11]. Apprehension about insufficient food access is a greater policy focus on the expenditure approach in achieving food security objectives [5]. From Econometric point of view, most researchers in the above stated studies employed only Propensity Score Matching (PSM) and ordinary least squares(OLS) methods, but the results from propensity score could not show the real adoption impact of agricultural technologies because the analytical tool failed to account self-selection arose from unobserved endogeneity problem [16]. In addition, there is a scanty of empirical evidence which illustrates the impact and status of improved *teff* varieties developed, disseminated towards the rural farmers by the nearby research center and its attribution in enhancing food security and livelihood outcomes for smallholder farm households This research work contributes to the existing literature by providing empirical evidence on the impact of ITVs on food security by taking in to account selection biased stemmed from both observed and un observed heterogeneity using endogenous switching regression to generate a consistent and unbiased estimate on expenditure dimension of food security. Thus, to better inform future research and policy, this study attempts to evaluate the impact of ITVs adoption on food security measured by daily kilocalorie available per adult equivalent and annual food consumption expenditure per adult equivalent.

## 2. Literature review

Different scholars undertake research on factors influencing the adoption of improved agricultural technologies and evaluate the impact of adoption of agricultural technology on food security in Ethiopia and abroad where the agriculture sectors are the engine of development which drives the entire economy. Numerous studies conducted on the impact of adoption of improved crop varieties and other accompanying technologies showed a positive attribution for smallholder farmer’s food security measured by income, household expenditure, daily calorie intake, food consumption score, and dietary diversity generally on food security of farm households using different food security indicators. For example Hailu *et a*l. (2021) analyzed the impacts of agricultural technology adoption on household food security among smallholder farm households using an endogenous switching regression model, the model result shows that the adoption of improved agricultural technologies significantly increases household caloric intake, dietary diversity, and food consumption scores [17]. Belay and Mengiste (2021) studied on adoption and impact of agricultural technology adoption on poverty reduction in Ethiopia using ESR. The results showed that farm household’s decisions to adopt agricultural technologies are determined by the sex of the household head, accessibility of credit service, amount of savings, number of extension visits, membership of farm cooperatives and distance from the market. Moreover, the result of ESR model revealed that the adoption of agricultural technology had significant impact on increasing household consumption expenditure and in reducing household poverty status [18]. Etsehiwot and Solomon (2020) examine factors influencing agricultural technology adoption and its’ impact on *teff* productivity in Ethiopia using endogenous switching regression model. The result indicates that technology adoption is positively determined by sex, education, soil fertility status, frequency of extension contact by development agent visits, whereas age, distance to main road, distance to the input and output market, parcel ownership and farm size influence negatively and significantly. The result of endogenous switching regression model found that adoption of improved *teff* has a positive and significant impact on *teff* productivity [13]. Regasa *et al*. (2018) conducted a research on the determinants of improved *teff* varieties adoption and its impact on productivity in the case of non-traditional *teff* growing areas of Western Ethiopia using propensity score matching. The results shows that dependency ratio, land allocated to cereal crops other than *teff* and horticultural crops had negative and significantly whereas livestock ownership, access to training and information on *teff*, status of the household heads (being progressive farmer) and being improved *teff* cultivars of friends, relatives and neighbors have contributed positively and significantly to *teff* adoption in the study areas. The results also found that the adoption of improved *teff* has a positive and significant impact on *teff* productivity [15].A study conducted by Bekele *et al*. (2014) on adoption and the impact of improved wheat on food security using both propensity score matching and endogenous switching regression methods of impact evaluation in Ethiopia. The finding affirms that education, access to input and output market, variety information, agro-ecology, input access, extension contact positively whereas price of competing crops negatively affects the decision to adopt improved wheat variety. The result of the propensity score matching and ESR indicate that adoption of improved wheat varieties significantly increases the probability of food security, per capita food consumption, and the probability of attaining the food breakeven and food surplus status [4]. Tsegaye and Bekele (2012) studied on the determinants of adoption of improved wheat technologies and its impact on food consumption in Ethiopia using propensity score matching. The results shows confirms that age of household head, education level, farm experience, participation in off-farm activities, access to credit, extension contact, and livestock holding a determine farmers’ adoption decision of wheat technologies. The finding of the impact result revealed that the food consumption per adult equivalent per day for adopters of improve wheat variety was higher for adopters than non-adopters of the improved variety [7]. Debelo (2015) assess the determinant factors to adopt Kuncho *teff* variety and its impact on crop net income using propensity score matching in Ethiopia. The results indicates that family labor availability, participation of farmers in agricultural trainings, education level of the household head, livestock holding and frequency of extension contact were the determinant factors which positively affect decision to adopt Kuncho *teff*. On the contrary age of household head, owning oxen and distance from household residence to market center was found adversely influence adoption of Kuncho *teff*. Furthermore, adoption of Kuncho *teff* on farmers’ significantly increases crops income [12]. Jaleta *et al*. (2018) studied the impact of improved varieties on food consumption expenditure using endogenous switching regression model and found that adoption of improved technologies had a significant impact on food consumption per adult equivalent per day [2].Belay *et a*l. (2022) studied on the impact of agricultural technologies on food consumption expenditure in Amhara Region, Ethiopia The result shows that adopting agricultural technology significantly increases household food consumption expenditure per adult equivalent [19].

## 3. Materials and methods

### 3.1. Description of the study area

This study was conducted in Lasta Woreda, one of the districts of North Wollo Zone which is found inside Tekeze basin growth corridor of Amhara National Regional State, Ethiopia. The district is located in the northeastern part of Amhara National regional state at 13°20’ N’ latitude and 38o58’ E’ longitude. The Woreda is bordered by Wagehimera zone in the North [20].

The woreda has 24 rural administrative kebeles with a total population of 119, 482. The altitude of the woreda ranges from 1400 to 4200 meter above sea level with four agro climatic zones of which 48.1% Woina dega, 38% kola, 15.4% dega 0.2% frost. The area receives an average annual rainfall of 533–880 millimeters and minimum and maximum temperatures of 16°C and 27°C, respectively. Lasta woreda is characterized by food insecurity and drought prone areas of the Amhara region, Ethiopia. The livelihood of this population relays on mixed farming dominated by crop production About 4,000 ha of land in the woreda are cultivated during Belg season while 28,071 ha of land is arable land in the main season and 3105 hectares of land is cultivated through irrigation. Among the different crops grown in the area, *teff* is the dominant crop and grown in 18 kebeles among 24 administrative kebeles in the Woreda. Wheat, barley and sorghum are the dominant crops grown by smallholder farm households in the Woreda [21].

### 3.2. Data and sampling procedure

Primary data were collected through a formal interview of sample households using semi-structured questionnaire. The primary data were collected by well-trained enumerators under the supervision and coordination of the researcher. Secondary data were also collected from the secondary data sources by reviewing different published and unpublished articles, proceedings, and books. Multi-stage sampling techniques were used to select representative sample households for this study. First, 13 major *teff* producer kebeles were selected purposively where ITVs were introduced among 23 kebeles of Lasta Woreda. Secondly, three kebeles were randomly selected from 13 *teff* producing kebeles. In the third stage, households in the selected three kebeles were stratified into two groups of adopters and non-adopters of ITVs. Finally, using a simple random sampling technique, from each kebeles in each category proportional to the total number of sample households, 225 sample households were selected randomly (119 from adopters and 106 non-adopters).

Data were collected on both standard daily kilocalorie intake and subjective food security indicators. The standard indicator of household food security, daily kilocalorie intake, was estimated through a direct survey of actual daily kilocalorie availability. For this analysis, the daily kilo caloric acquisition of households was used as an outcome variable to assess food security. The volume of each food item consumed in kilograms or liters in the study area was obtained from seven days recall period before the survey day that respective household’s consumed from their own produce, through purchases and aid in the study area. Then, weekly consumption data was converted into kilocalorie using the nationally standardized food composition table manual. The converted data were divided into household adult equivalents. Following this, it was converted to kilo calorie per day basis. Moreover, subjective food security indicators using respondents perception on food security status (whether households faced food shortage throughout the year or not), following [4] to complement with -objective measurement indicators.

### 3.3. Data analysis methodology

The collected data were analyzed using both descriptive statistics and econometric models.

#### 3.3.1. Descriptive and inferential statistics

The data which were collected from sample households was analyzed by using descriptive statistics such mean, minimum, maximum, standard deviation, and percentages. Moreover, test statistics such as t-test was applied for discrete and continuous variables and chi-square (χ2) test was also applied for dummy and categorical variables to check the significance of variables between adopters and non-adopters of ITVs.

#### 3.3.2. Endogenous switching regression (ESR)

To estimate the decision to adopt improved teff varieties (ITVs) and its impact on food security, two stage endogenous switching regression (ESR), which accounts both the observed and an observed heterogeneity effect was used. In the first stage of ESR, farmers’ decision to adopt improved *teff* varieties is modeled and estimated using a probit model. Following a random utility maximization theory which coincides with empirical literatures [2, 4], farmers adopt ITVs if the perceived benefit from adoption of ITVs is greater than its counterpart. Consider the *k*^*th*^ farm households decision to adopt or not. Let *U*_0_ represent the expected benefits from non-adoption of ITVs, and let *U*_*k*_ represent the benefit generated from adoption of ITVs. If we assume that the latent variable K_i_* (U_k-U0_) represents the utility (the benefit from adoption), the farmer will adopt improved technology if $K_{i}^{*}=U_{k}^{}-U_{0}>0$. The net benefit $\left(K_{i}^{*}\right)$ cannot be observed and is a latent variable, which is a function of observed characteristics (*z*_*i*_) and the error term (*ε*_*i*_):

$$K_{i}^{*}=z_{i}\alpha +\varepsilon_{i}\;\mathrm{With}\;K_{i}=\{\begin{array}{l} 1\;\mathrm{if}\;K_{i}^{*}>0\\ 0\;\mathrm{otherwise} \end{array}$$ (1)

Where *K*_*i*_ is a binary indicator variable (latent variable) that takes a value 1 if a farmer is an adopter of ITVs and zero otherwise and *α* is a vector of parameters to be estimated. In this study, adoption is defined as farmers who used any of the ITVs, either newly purchased from primary cooperatives or reuse of ITVs from previous year production.

In the second stage, conditional on the selection equation, the outcome variables can be written as the switching regime following [22] as follows:

$$\mathrm{Regime}\;1:\;y_{1i}=\beta_{1}X1i+\eta_{1i},\;if\;K=1$$ (2A)

$$\mathrm{Regime}\;2:\;y_{2i}=\beta_{2}X2i+\eta_{2i},\;if\;K=0$$ (2B)

Where y_1i_ and y_2i_ are outcome variables for adopters and non-adopters of ITVs, X_i_ is

Vector of observed socio-economics and farm level characteristics, β is a vector of parameters to be estimated.

For the ESR model to be identified, it is important to apply exclusion restrictions as a selection instrument that directly determines the selection equation or the decision to adopt but not the outcome equation. In this study, a number of seed traders known by farmers and sources of ITVs, information from development agents and research centers are used as instrumental variables for the impact of adoption of ITVs on the outcome variables of interest for this study. Adoption characteristics of smallholder farmers can be determined by the availability of different information sources and those information sources used as a tool for facilitating dissemination process of agricultural technologies. Similarly, the number of local seed traders/buyers influences the local ease accessibility of seed and determines farmers to adopt improved technologies [2, 4].

A simple falsification test following [2] was done to test the validity of the instruments which significantly determine the selection equations or adoption decisions but not the outcome variables among farm households that did not adopt ITVs. As shown in S1 Appendix part of the supporting information for both daily caloric intake and food consumption expenditure, which clearly illustrate that that access to variety information and the number of seed traders known by farmers are used as valid selection instruments for t outcome variables used for this study: they are jointly statistically significant in the selection equation (χ 2 = 102.85; p = 0.00) but not for the outcome equation for the farm households that did not adopt ITVs. (F-stat. = 1.79, p = 0.1542 and F-stat. = 0.5, p = 0.6833) for daily caloric intake and annual food consumption expenditure per adult equivalent outcome variables respectively, whereas for binary food security status [χ 2 = 1.23 (p = 0.3023) which verified the validity of the instruments used for this study (see Tables 1 and 2 in S1 Appendix in the supporting information part).

The stochastic terms in Eqs (1) and (2) are assumed to have a trivariate normal distribution with mean zero and non-singular covariance matrix specified as:

$$\mathrm{cov}(\varepsilon ,\eta_{1},\eta_{2})=[\begin{array}{l} \sigma_{\varepsilon }^{2}\;\sigma_{\varepsilon 1}\;\sigma_{\varepsilon 2}\\ \sigma_{1\varepsilon }\;\sigma_{1}^{2}\;.\\ \sigma_{2\varepsilon }\;.\;\sigma_{2}^{2} \end{array}],$$ (3)

Where $\sigma_{\varepsilon }^{2}=\mathrm{var}(\varepsilon ),\sigma_{1}^{2}=\mathrm{var}(\eta_{1}),\sigma_{2}^{2}=\mathrm{var}(\eta_{2}),\sigma_{\varepsilon 1}=\mathrm{cov}(\varepsilon ,\eta_{1}),\mathrm{and}\;\sigma_{\varepsilon 2}=\mathrm{cov}(\varepsilon ,\eta )$. It is plausible that the variance of $\sigma_{\varepsilon }^{2}$ is assumed to be equal to 1 because the *β* coefficients in the selection model are estimable up to a scale factor. The covariance between *η*_1_ and *η*_2_ is not defined since y_1_ and y_2_ are not observed simultaneously [22].The expected values of *η*_1_ and *η*_2_ conditional on the sample selection is non-zero due to the fact that the error term in the selection Eq (1) is correlated with the error terms in Eqs (2A) and (2B) respectively. The expected value of the error terms in Eqs (2A) and (2B) can be expressed as follows:

$$E(\eta_{i}{}_{1}|K_{i}=1)=\sigma_{1\varepsilon }\frac{\phi (z_{i}\alpha )}{\Phi (z_{i}\alpha )}\;=\sigma_{1\varepsilon }\lambda_{i1}\;And,$$

$$E(\eta_{i}{}_{2}|K_{i}=0)=-\sigma_{2\varepsilon }\frac{\phi (z_{i}\alpha )}{1-\Phi (z_{i}\alpha )}\;=\sigma_{2\varepsilon }\lambda_{i2}$$

Where *ϕ*(.) is the standard normal probability density function, Φ(.) is the standard normal cumulative density function, $\lambda_{i1}=\frac{\phi (z_{i}\alpha )}{\Phi (z_{i}\alpha )}$ and $\lambda_{i2}=\frac{\phi (z_{i}\alpha )}{1-\Phi (z_{i}\alpha )}$. Where *λ*_*i*1_ and *λ*_*i*2_ are the Inverse Mills Ratios (IMR) estimated from the selection equation and then will be incorporated in Eqs (2A) and (2B) to correct for selection bias in a two-step estimation procedure(ESR).

*Conditional expectations*, *treatment and heterogeneity effects*. The above framework can be used to estimate the average treatment effect on the treated (ATT) and the average treatment effect on untreated (ATU) by comparing the anticipated values of the outcomes for adopters and non-adopters in actual and counterfactual scenarios following [23] defined as follows:

Adopters with adoption of ITVs (observed scenario)

$$E(y_{i1}|K=1;x)=x_{i1}\beta_{1}+\sigma_{1\varepsilon }\lambda_{i1}$$ (4A)

Non-adopters without adoption (observed scenario)

$$E(y_{i2}|K=0;x)=x_{i2}\beta_{2}+\sigma_{2\varepsilon }\lambda_{i2}$$ (4B)

Non-adopters had they decided to adopt (counterfactual scenario)

$$E(y_{i2}|K=1;x)=x_{i1}\beta_{2}+\sigma_{2\varepsilon }\lambda_{i1}$$ (4C)

Adopters had they decided not adopt (counterfactual scenario)

$$E(y_{i1}|K=0;x)=x_{i2}\beta_{1}+\sigma_{1\varepsilon }\lambda_{i2}$$ (4D)

The average treatment effect on the treated (ATT) is estimated as the difference between (4a) and (4c), which is the impact of adoption of ITVs on the outcome of interest for adopters.

$$ATT=E(y_{i1}|K=1,x)-E(y_{i2}|K=1,x)=x_{i1}(\beta_{1}-\beta_{2})+\lambda_{1i}(\sigma_{1\varepsilon }-\sigma_{2\varepsilon })$$ (5)

Similarly, the expected change in non-adopter’s food security, the effect of the treatment on the untreated (ATU) is computed as the difference between (4d) and (4b)

$$ATU=E(y_{i1}|K=0,x)-E(y_{i2}|K=0,x)=x_{i2}(\beta_{1}-\beta_{2})+\lambda_{2i}(\sigma_{1\varepsilon }-\sigma_{2\varepsilon })$$ (6)

The heterogeneity effect is the difference between Eqs (4A)–(4D)

Following [24], the base heterogeneity effect (BH_1_) for farmers that decided to adopt ITVs is estimated as the difference of Eqs (4A) and (4D),

$$\begin{array}{l} BH_{1}=E(y1i|Ai=1)-E(y1i|Ai=0)\\ \;=(X1i-X2i)\beta_{1i}+\sigma 1\eta (\lambda 1i-\lambda 2i). \end{array}$$ (7)

In the same manner, the base heterogeneity (BH_2_) for farmers that decided not to adopt the ITVs is the difference between Eqs (4C) and (4B),

$$\begin{array}{l} BH_{2}=E(y2i|A_{i}=1)-E(y2i|Ai=0)\\ =(X1_{i}-X_{2}i)\beta_{2i}+\sigma 2\eta (\lambda 1i-\lambda 2i). \end{array}$$ (8)

Lastly, the transitional heterogeneity (TH) is the difference between Eqs (5) and (6).

## 4. Results and discussion

### 4.1. Mean difference of explanatory variables between adopters and non-adopters

As presented in Table 1, the explanatory variables used in adoption decision and impact analysis disaggregated by adopters and non-adopters of ITVs. Accordingly, the proportion of male-headed and female-headed sample respondents is 96% and 4%, respectively. The result indicates that among the total adopters, 98.3% were male headed households and the remaining 1.7% was female headed households. Among the total non-adopters, 92.4% were male headed households and the remaining were female headed smallholder farm households. The mean total family size measured in adult equivalent for adopters was 4.345, while it was 3.88 for non-adopters and the result shows a significant mean difference between adopters and non-adopters smallholder farm households. Concerning the total livestock units, the mean livestock owned in log form (TLU) by adopters was 1.431, whereas the mean livestock holding for those non-adopters of ITVs was 1.243 measured in the tropical livestock units and the mean difference is statistically significant at the 5% probability level.

**Table 1:** Mean difference in explanatory variables between adopters and non- adopters.

Variables	Adopters	Non-Adopters	difference
Sex(%)of household head male(male = 1)	0.983	0.924	4.54**
Age of household head in years	48.915	47.433	-1.047
Age square of household head in years	2479.15	2336.96	-1.029
Year of schooling	2.579	2.084	-1.225
Membership in farmers cooperatives(%yes)	0.605	0.377	11.62***
Total family size in adult equivalent	4.345	3.885	-2.96***
Total farm land owned by household in hectare	0.759	0.696	-1.252
Log livestock owned in Tropical livestock unit(TLU)	1.431	1.243	-2.96***
Off-farm income in Ethiopia Birr	2646.63	1192.972	-2.82***
Frequency of extension visit	2.815	1.669	-4.57***
Provision of training(%yes)(yes = 1)	0.714	0.594	3.58*
Access and need of credit service(%yes)(yes = 1)	0.058	0.009	3.988**
Number of seed traders known by respondents	3.176	1.009	-7.55***
Variety information from development agents(%yes)(yes = 1)	0 .907	0.547	37.63***
Variety information from research center(%yes)(yes = 1)	0.336	0.132	12.78***
Distance from respondents residence to Kebele office of agriculture in kilometer.	1.968	2.748	4.57***

*, ** and *** denote the significance level at 10%, 5%, and 1% probability level, respectively.

Furthermore, among the total adopters of improved *teff* varieties 90.7% and, among the total non-adopters of improved t*eff* varieties, 54.7% had access to variety information from development agents.

### 4.2. Factors affecting adoption of ITVs

The result from the Probit model indicates that the determinant factors which determine farmer’s adoption decision of ITVs in this study are total family size, off-farm income, number of traders known by farmers, variety information from development agents and research center affects positively whereas the distance of farmer’s residence from kebele office of agriculture negatively and significantly determine farmer’s decision to use ITVs.

As depicted in Table 2, family size measured in adult equivalent is one of the determinant factors which determine the adoption decision of ITVs in smallholder farm households. Family size positively determines the use/adoption of ITVs. The reason might be ITVs require more labor during the critical time to implement basic agronomic practices such as plowing, sowing, weeding and harvesting, especially the adoption of improved *teff* variety absorbs more labor force if farmers practice row planting and fertilizer application and other necessary complimentary packages during sowing. The Probit model affirms that a unit increase in family size results in the likelihood of adopting ITVs increases by 11.19%. The result is coinciding with the adoption literature [3].

**Table 2:** Factors affecting the decision to adopt ITVs probit model.

Explanatory variable	coefficient	Robust St. error	Marginal effect
Sex of household head(male = 1)	0.911	0.592	0.332
Age of household head	-0.088	0.106	-0.035
Age square of household head	0.001	0.001	0.0004
Year of schooling	0.032	0.041	0.012
Membership in farmers cooperatives	0.0374	0.237	0.014
Total family size	0.2812***	0.100	0.111
Total farm land owned	-0.4349	0.339	-0.173
Log Livestock ownership in TLU	0.236	0.319	0.094
Off-farm income	0.00009**	0.00004	0.00003
Frequency of extension visit	0.097	0.068	0.038
Provision of training	0.033	0.249	0.013
Access and need of credit service	-0.791	0.714	-0.295
Number of seed traders known by respondents	0.274***	0.059	0.109
variety information from development agents	1.811***	0.329	0.604
variety information from research center	0.659***	0.248	0.251
Distance from kebele office of agriculture	-0.191**	0.094	-0.076
Number of observations	225		
Wald chi2	87.36		
Prob > chi2	.000		
Pseudo R2	0.487		
log likelihood	79.721		

*, ** and *** denote the significance level at 10%, 5%, and 1% probability level, respectively.

Variety information from development agents and research centers are determinant factors which determine the probability of adopting ITVs. Availability of information on improved *v*ariety production and variety parameters such as productivity, earliness, disease and pest resistant, lodging behavior and other variety traits information from development agents and researchers in the study area had a positive effect on the adoption decision of smallholder farmers ITVs. Access to information is supposed to make sure confidence on updating the nature of the available technologies and farmers to have a better understanding of the variety trait and encourages adopting improved technologies. The result of the marginal effect of probit model shows that those farm households who have got variety information from development agents and research center increases the probability of adopting improved *teff* by 60.47% and 25.12% more than their counter part respectively. This result is consistent with the result of [13, 25–27].

Distance of farmer’s residence from Kebele office agriculture is the other limiting factor which negatively influences the adoption decision of ITVs. The result of the marginal effect from the binary probit model revealed that as the distance of farmer residence from Kebele office of agriculture increases by one kilometer, the probability to adopt ITVs decreases by 7.63% at the 1% probability level. This might be due to limited access to information on improved varieties, as farmers are far from the office of the respective Keble agriculture office. The reason might be the extension service providers which are found in the study area might not reach those farmers who are far away from their respective office of agriculture on frequent provision of extension service and awareness creation on the importance of the adoption of the existing improved *teff* varieties. This result is consistent with the results of literatures done on adoption and impact studies [13, 26, 27]. Our findings also show that off-farm income is important for the adoption of ITVs. The justification for this finding is that the income generated by engaging in off-farm activities solves liquidity problems by offsetting the financial shortfall of the farm households to purchase improved seeds, other complementary inputs necessary for *teff* production and might hire other labor source to apply the full. This result is parallel with the previous empirical literatures [18, 28, 29]. Number of seed traders known by respondents is also other determinant factor for adoption of ITVs. Because the presence of local seed traders in locality increase the probability of getting ITVs for the local community and helps to use improved t*eff* varieties.we found similar result where the number of seed traders known by sampled households determine adoption of improved agricultural technologies [4].

### 4.3. Impact of adoption of ITVs on food security using ESR

The results from ESR model estimated by full information maximum likelihood revealed that the estimated error correlation between the selection equation and outcome equations for adopters for both outcome variables (daily caloric intake and food consumption expenditure) is negative for both groups and are significantly different from zero only for the correlation between the selection equation and for both outcome equations for adopters rho1 (ρj). The results indicate that both observed and unobserved factors influence the decision to adopt ITVs and outcome variables.

The significance of the coefficient of correlation between the adoption equation and the outcome variables demonstrates that there is sample selectivity bias in the adoption of ITVs and illustrates the existence of heterogeneity in the sampled households as shown in S2 Appendix part of the supporting information for both daily kilo calorie intake per adult equivalent, food consumption expenditure per year per adult equivalent in Ethiopian birr.

The likelihood-ratio tests for joint independence of the three equations are reported in the last lines of S2 Appendix part of the supporting information (LR χ2 = 3.73, p = 0.0536) and (LR χ2 = 6.07, p = 0.013) for daily calorie intake and food consumption expenditure per adult equivalent respectively. The test show that the error term of the selection Eq (1), the error terms of equation outcome Eqs (2A) and (2B), are correlated and overlooking them leads to biased results(see Tables 1 and 2 in S2 Appendix in the supporting information).

Full information maximum likelihood ESR model results as depicted in Table 3 clearly shows that adoption of ITVs had a positive and significant effect on food security measured by daily kilocalorie intake and food consumption expenditure per adult equivalent. Had the adopter households decided not to adopt ITVs, their average daily calorie intake per adult equivalent would have decreased by 417.908 kilocalorie, which is significant at the 1% probability level. On the other hand, if non-adopter households would have adopted ITVs, their daily calorie intake per adult equivalent would have increased by 512.581 kilocalories which is higher than the benefit adopters would have lost due to non-using or non-adoption of ITVs (when adopters decide not to adopt).

**Table 3:** Average treatment effect on treated (ATT) of daily calorie intake per adult equivalent using ESR.

a)Daily caloric intake PAE	Decision stage		Treatment effect
	Adopted	Not to adopt	
Adopters	2026.420	1608.512	417.908***
	(53.418)	(50.565)	(73.555)
Non-adopters	2126.713	1614.132	512.581***
	(61.167)	(45.033)	(75.957)
Heterogeneity effects	BH1 = -100.293	BH2 = -5.620	TH = -94.673

Standard error in parenthesis; ***, denotes the significance level at 1% probability level.

Furthermore, for the binary food security variable as depicted in Table 5, the average probability of being food secure decreases by 20.1 percentage points for adopters of ITVs had they decided not to adopt the improved varieties In the same way, the average probability of food security increases by 30.3% points for non-adopters had they adopted ITVs.

Base heterogeneity (BH1) is negative for daily calorie intake per adult equivalent indicating that if the non-adopters had been adopted, they would have got significantly higher daily kilo calorie intake per adult equivalent (about 100.293 kilocalories (4.949%) more) than the actual adopters of ITVs. Base heterogeneity effect (BH2) indicates that adopters would have got less daily calorie intake per adult equivalent than the actual non-adopters if they had not been adopted. The transitional heterogeneity effects (TH), which illustrates whether the impact of adopting ITVs is larger or smaller for farm households that adopted ITVS or for farm households that did not adopt, had they did adopt the ITVs. The result of the transitional heterogeneity effect for both daily calorie intake and consumption expenditure per adult equivalent is positive, which implies that non-adopters would have benefited more if they decided to adopt the ITVs than the actual impact of adopters due to adopting of the improved variety, Which means the ATT of adopters is significantly smaller for actual adopters compared to actual non-adopters if non-adopters decide to adopt ITVs. We found a similar results where the adoption of improved technologies had a significant impact on food security measured by different food security indicators [4, 13, 14, 17].

Regarding the average annual food consumption expenditure as shown in Table 4, had the adopter households decided not to adopt the ITVs, their average food consumption expenditure per adult equivalent per year would have decreased by 1096.509 Ethiopian Birr. Moreover, if non-adopter households decided to adopt ITVs, their food consumption expenditure per adult equivalent would have increased by 1323.987 Ethiopian Birr. Accordingly, non-adopter farm households would be more benefited than adopter households if they switch to adopt the ITVs (Table 4). We found similar results where the adoption of improved technologies had a significant impact on food security measured by different food security indicators [2, 14, 17–19].

**Table 4:** ATT of food consumption expenditure using endogenous switching regression.

b)Food consumption expenditure PAE	Decision stage		Treatment effect
	Adopted	Not to adopt	
Adopters	4929.360	3832.850	1096.509***
	(195.123)	(94.819)	(216.942)
Non-adopters	4732.527	3408.540	1323.987***
	(310.711)	(81.775)	(321.292)
Heterogeneity effects	BH1 = 226.856	BH2 = 468.682	TH = -241.826

***denotes significance level at 1% probability level, standard error in parenthesis.

Furthermore, when we look at the binary food security variable as depicted in Table 5, the probability of being food secure status decreases by 20.1 percentage points for users of ITVs had they decided not to improve ITVs. Similarly, the probability of food security status increases by 30.3% points for non-adopters had they decided to use/to adopt ITVs as indicated in Table 5.

**Table 5:** Average treatment effect on the probability of food security.

c) probability of food security	Decision stage		Treatment effect
	Adopted	Not to adopt	
Adopters	0. 697	0.496	0. 201***
Non-adopters	0.642	0.338	0.303***

***, denotes significance level at 1% probability level

## 5. Conclusions and recommendations

This article examines adoption and impact of improved teff varieties adoption on food security measured by daily caloric intake and food consumption expenditure. The result of this study shows that the adoption decision of improved *teff* varieties is determined by total family size measured in adult equivalent, off-farm income, number of traders known by farmers, variety information from development agents and research center positively and distance of farmer’s residence from kebele office of agriculture negatively. The adoption of ITVs have a positive and significant impact on food security measured by daily kilo caloric intake per adult equivalent and annual food consumption expenditure in the study area. Therefore, to enhance the food security of smallholder farmers, agricultuar extenstion service providers should facilitate acess to information for smallholder farmers about ITVs by cosidering those farmers far from the office of development agents in order to facilitate diffustion of ITVs and promote those local seed traders either newly purchased or reused inorder to increase the availablity of improved seed for the local community.From the research perspective, to strength further diffusion of ITVs and to extricate smallholder farmers from food insecurity due attension should given on multiplication of basic improved *teff* seeds which are already released,developing new improved varieties and development of labour saving technologies because labor is one of the limiting factor which detemine the adoption of ITV in study area. From government point of view, agricultural policies and strategies designed to realize food security in the study are should take in to account promopting of ITVs.

## Supporting information

S1 Appendix Test on the validity of the selected instruments for outcome variables. (DOCX)

S2 Appendix Endogenous switching regression estimate for outcome variables. (DOCX)

S1 Data (DTA)
