# Supplementary material for: Adoption and impact of improved teff varieties adoption on food security: Micro level evidence from North Eastern Amhara Regional state, Ethiopia
Source: PLoS One. 2023 Sep 20;18(9):e0291434. doi: 10.1371/journal.pone.0291434 (PMC10511098; doi:10.1371/journal.pone.0291434)
Supplement: S1 Appendix — (DOCX) [file pone.0291434.s001.docx]

**Appendix Table1. Test on the validity of the Selected Instruments for daily calorie intake**

| **Instrumental variable** | **Adoption decision (1=yes)** | **For non-adopters** |
| --- | --- | --- |
| Variety information from development agents | 1.519***  ( 0 .256) | -33.045  (128.898) |
| Variety information from research center | 0.682***  (0.240) | 23.445  (184.330) |
| Number of seed traders known by farmers | 0.304*** (0.044) | 64.606 **  ( 29.754) |
| test | *χ* 2 =102.85*** | F-stat. = 1.79 |
| sample | 225 | 106 |

*Note*: for adoption decision: Probit model; for outcome variable: ordinary least squares. Standard errors in parenthesis; **^,^ and *** denotes significance level at 5% and 1% respectively.

**Appendix Table 2. Test on the Validity of the Selected Instruments for consumption expenditure**

| **Instrumental variable** | **Adoption decision (1=yes)** | **For non-adopters** |
| --- | --- | --- |
| Information from development agents | 1.519***  ( 0.256) | -131.786  (268.589) |
| Variety information from research center | 0.682 ***  (0.240) | 343.828  (384.095) |
| Number of seed traders known by farmers | 0.304***  (0.044) | -50.1026  (62.001) |
| test | *χ* 2 =102.85 *** | F-stat. = 0.5 |
| sample | 225 | 106 |

Standard errors in parenthesis; *** denotes significance level at 1% probability level
