# Supplementary material for: Adoption and impact of improved teff varieties adoption on food security: Micro level evidence from North Eastern Amhara Regional state, Ethiopia
Source: PLoS One. 2023 Sep 20;18(9):e0291434. doi: 10.1371/journal.pone.0291434 (PMC10511098; doi:10.1371/journal.pone.0291434)
Supplement: S2 Appendix — (DOCX) [file pone.0291434.s002.docx]

**Appendix Table 1. Endogenous switching regression estimate for daily calorie intake**

| **Variables** | | **Adopters** | | | **Non-Adopters** | |
| --- | --- | --- | --- | --- | --- | --- |
|  | **Coef.** | | **St. error** | **Coef.** | | **St. error** |
| Sex of household head | 231.696** | | 367.197 | -185.423 | | 183.002 |
| Age of household head | -394.35*** | | 46.193 | 16.764 | | 15.287 |
| Age square of household head | 56.925 | | 0.444 | -0.156 | | 0.144 |
| Year of schooling | 467.323*** | | 16.579 | 1.059 | | 18.047 |
| Membership in farmers cooperatives | -121.992 | | 100.951 | 80.458 | | 98.969 |
| family size in adult equivalent | 0.0176* | | 41.563 | -396.72*** | | 50.064 |
| Land holding size | -30.169 | | 137.258 | 111.437 | | 138.952 |
| Log of tropical livestock holding | -181.976 | | 127.992 | 148.224 | | 126.008 |
| Off-farm income | -12.422 | | 0.010 | 0.0008 | | 0.022 |
| Frequency of extension visit | 43.113 | | 25.719 | 63.085** | | 28.271 |
| Provision of training | 231.696** | | 117.127 | 93.902 | | 100.990 |
| Access to credit service | -394.35*** | | 202.145 | 4.672 | | 493.918 |
| Distance from kebele office | 56.925 | | 44.788 | -68.0780** | | 34.609 |
| constant | 467.32*** | | 1651.387 | 1625.823** | | 810.585 |
| Sigma(*σi*) | 493.7979 | | 34.753 | 454.154 | | 31.766 |
| Rho(*ρj*) | -0.0429* | | 0.198 | -0.1401 | | 0.3645 |
| Wald Chi2 | 163.28 | | | | | |
| Log likelihood | -1780.6223 | | | | | |
| Number of observations | 225 | | | | | |
| LR test of independency | chi2 = 3.73 Prob > chi2 = 0.0536 | | | | | |

*^,^ ** and *** denotes significance level at 10% ,5% and 1% respectively

**AppendixTable2. Endogenous switching regression estimate of food consumption expenditure per adult equivalent**

| Food consumption Expenditure per adult equivalent | | | | | |  |
| --- | --- | --- | --- | --- | --- | --- |
| Variables | **Adopters** | | **Non-Adopters** | |  |  |
|  | **Coef.** | **St. error** | **Coef.** | **St.error** | | |
| Sex of household head | 512.702 | 1298.3 | 720.221 | 408.69 | | |
| Age of household head | -71.785 | 163.38 | -0.393 | 34.53 | | |
| Age square of household head | 0.677 | 1.571 | 0.088 | 0.32 | | |
| Year of schooling | -28.264 | 58.825 | -92.520 | 40.57 | | |
| Membership in farmers  cooperatives | 135.041 | 357.70 | -321.88 | 223.4 | | |
| Total family size in adult equivalent | -16.43*** | 147.27 | -326.6*** | 111.4 | | |
| Land holding size | -507.385 | 486.62 | 678.978** | 314.6 | | |
| Log TLU | -74.696 | 452.99 | 92.398 | 280.6 | | |
| Off-farm income in ETB | 0.059 | 0.037 | -0.048 | 0.049 | | |
| Frequency of extension visit | -148.78* | 89.878 | 43.370 | 63.57 | | |
| Provision of training | -256.100 | 414.5 | 367.721 | 227.32 | | |
| Access to credit service | -556.440 | 717.81 | 2820.07** | 1102.9 | | |
| Distance from kebele office of agriculture | -350.7** | 156.99 | -179.212** | 76.30 | | |
| constant | -17929.3 | 5776.7 | --220.1829 | 1811. | | |
| Sigma | 1756.741 | 123.08 | 1025.42 | 71.09 | | |
| Rho(*ρj*) | -0.46*** | 0.153 | 0.1198 | 0.296 | | |

| Wald Chi^2^  168.890 |
| --- |
| Log likelihood -2016.347 |
| Number of observations 225 |
| LR test of independency: chi2 = 6.07 Prob > chi2 = 0.013 |

*^,^ ** and *** denote significance level at 10%,5% and 1% respectively
